# Transition between inpatient rehabilitation and National Disability Insurance Scheme for Traumatic Brain Injury and Spinal Cord Injury.

**DOI:** 10.23889/ijpds.v7i3.1864

**Published:** 2022-08-25

**Authors:** Simon Guthrie, Tara Alexander

**Affiliations:** 1 Macquarie University; 2 Australasian Rehabilitations Outcomes Centre

## Objectives

To understand the pathway and diverse levels of functional impairment for people with traumatic brain injury (TBI) and spinal cord injury (SCI) as they transition from inpatient rehabilitation (IR) hospital setting to ongoing care and support under the National Disability Insurance Scheme (NDIS) in Australia.

## Approach

The Australasian Rehabilitation Outcomes Centre (AROC) has data on almost every inpatient rehabilitation episode of care since 2002, including TBI and SCI. AROC data is de-identified with a statistical linkage key (SLK-581). The NDIS dataset contains identified administrative data on participants of the scheme from which the SLK-581 was derived. Datasets were restricted to TBI and SCI records and the SLK with key dates used to link records together. The linkage was done in multiple passes with different levels of information with each link being validated using secondary information relating to date of injury, date of admission and geographical location.

## Results

Over the period 2012-2019, approximately 2,000 records from AROC episodes were linked to an NDIS participant following data validation and individual review of borderline matches We will compare the functional independence of the individual upon leaving rehabilitation with their need for support under the NDIS. Functional independence in rehabilitation is measured by clinicians using the Functional Independence Measure (FIM), a tool that requires clinicians to be trained and credentialed in its use as it is part of the funding model for IR in Australia. Need for support under the NDIS is measured by the funded supports available to a participant under the plan. We expect to demonstrate a correlation between FIM scores and funded supports and identify and analyse any unexpected results.

## Conclusion and Relevance

These results will inform resource allocation within the NDIS. This project demonstrates how de-identified research datasets can be linked with administrative datasets to draw new and powerful insights into government service delivery and population health while maintaining privacy. Challenges to accurate linkage can be overcome through iterative and non-deterministic approaches.

